# Fisher Information as a Metric of Locally Optimal Processing and Stochastic Resonance

**DOI:** 10.1371/journal.pone.0034282

**Published:** 2012-04-06

**Authors:** Fabing Duan, François Chapeau-Blondeau, Derek Abbott

**Affiliations:** 1 College of Automation Engineering, Qingdao University, Qingdao, People's Republic of China; 2 Laboratoire d'Ingénierie des Systèmes Automatisés (LISA), Université d'Angers, Angers, France; 3 Centre for Biomedical Engineering (CBME) and School of Electrical & Electronic Engineering, The University of Adelaide, Adelaide, Southern Australia, Australia; University of Maribor, Slovenia

## Abstract

The origins of Fisher information are in its use as a performance measure for parametric estimation. We augment this and show that the Fisher information can characterize the performance in several other significant signal processing operations. For processing of a weak signal in additive white noise, we demonstrate that the Fisher information determines (i) the maximum output signal-to-noise ratio for a periodic signal; (ii) the optimum asymptotic efficacy for signal detection; (iii) the best cross-correlation coefficient for signal transmission; and (iv) the minimum mean square error of an unbiased estimator. This unifying picture, via inequalities on the Fisher information, is used to establish conditions where improvement by noise through stochastic resonance is feasible or not.

## Introduction

Fisher information is foremost a measure of the minimum error in estimating an unknown parameter of a probability distribution, and its importance is related to the Cramér-Rao inequality for unbiased estimators [1], [2]. By introducing a location parameter, the de Bruijn's identity indicates that the fundamental quantity of Fisher information is affiliated with the differential entropy of the minimum descriptive complexity of a random variable [1]. Furthermore, in known weak signal detection, a locally optimal detector, acting as the small-signal limited Neyman-Pearson detector, has favorable properties for small signal-to-noise ratios [3]. With sufficiently large observed data and using the central limit theorem, it is demonstrated that the locally optimal detector is asymptotically optimum and the Fisher information of the noise distribution is the upper bound of the asymptotic efficacy [2]–[7]. For weak random signal detection, the second order Fisher information is also associated with the maximum asymptotic efficacy of the generalized energy detector [4]–[7].

However, the fundamental nature of Fisher information is not adequately recognized for processing weak signals. To extend the heuristic studies of [1]–[7], in this paper, we will theoretically demonstrate that, for a weak signal buried in additive white noise, the performance for locally optimal processing can be generally measured by the Fisher information of the noise distribution. We show this for the following signal processing case studies: (i) the maximum output signal-to-noise ratio for a periodic signal; (ii) the optimum asymptotic efficacy for signal detection; (iii) the best cross-correlation coefficient for signal transmission; and (iv) the minimum mean square error of an unbiased estimator. The physical significance of Fisher information is that it provides a unified bound for characterizing the performance for locally optimal processing. Furthermore, we establish the Fisher information condition for stochastic resonance (SR) that has been studied for improving system performance over several decades [8]–[32]. In our recent work [28], it is established that improvement by adding noise is impossible for detecting a weak known signal. Here, based on Fisher information inequalities, we further prove that SR is not applicable for improving the performance of locally optimal processing in the considered cases (i)–(iv). This result generalizes a proof that existed previously only for a weak periodic signal in additive Gaussian noise [12], [33]. However, beyond these restrictive conditions, the observed noise-enhanced effects [9]–[11], [26], [28]–[30] show that SR can provide a signal processing enhancement using the constructive role of noise. The applications of SR to nonlinear signal processing are of practical interest.

## Results

In many situations we are interested in processing signals that are very weak compared to the noise level [2], [3], [6]. It would be desirable in these situations to determine an optimal memoryless nonlinearity in the following study cases.

### Output signal-to-noise ratio for a periodic signal

First, consider a static nonlinearity with its output
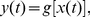
(1)where the function 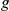 is a memoryless nonlinearity and the input is a signal-plus-noise mixture 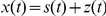. The component 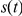 is a known weak periodic signal with a maximal amplitude 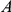 (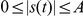) and period 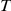. Zero-mean white noise 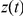, independent of 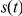, has probability density function (PDF) 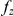 and a root-mean-square (RMS) amplitude 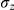. It is assumed that 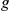 has zero mean under 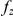, i.e. 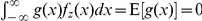, which is not restrictive since any arbitrary 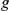 can always include a constant bias to cancel this average [6]. The input signal-to-noise ratio for 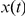 can be defined as the power contained in the spectral line 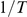 divided by the power contained in the noise background in a small frequency bin 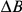 around 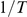 [10], this is
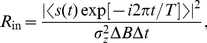
(2)with 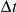 indicating the time resolution or the sampling time in a discrete-time implementation and the temporal average defined as 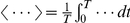 [10]. Here, we assume the sampling time 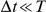 and observe the output 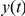 for a sufficiently large time interval of 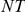 (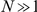) [10]. Since 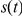 is periodic, 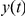 is in general a cyclostationary random signal with period 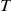 [10]. Similarly, the output signal-to-noise ratio for 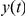 is given by
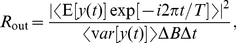
(3)with nonstationary expectation 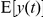 and nonstationary variance 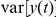 [10].

In the case of 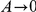, we have a Taylor expansion of the expectation at a fixed time 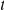 as
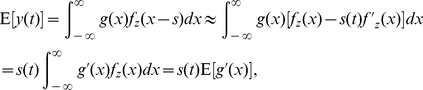
(4)where we assume the derivatives 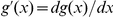 and 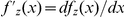 exist for almost all 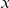 (similarly hereinafter) [2], [6]. Thus, we have
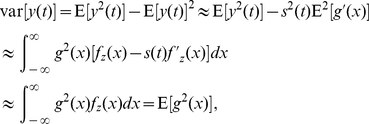
(5)where 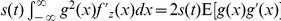 and 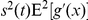, compared with 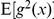, can be neglected as 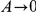 (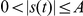) [2], [6]. The above derivations of Eqs. (4) and (5) are exact in the asymptotic limit for weak signals, and have been generally adopted in [2], [6].

Substituting Eqs. (4) and (5) into Eq. (3), we have
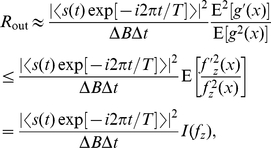
(6)where the expectation 
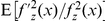
 is simply the Fisher information 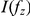 of the noise PDF 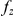 [2], [6], and the equality occurs as
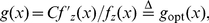
(7)by the Cauchy-Schwarz inequality for a constant 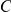 [2], [6].

Noting Eqs. (2) and (6), the output-input signal-to-noise ratio gain 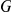 is bounded by
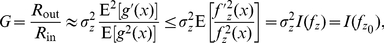
(8)with equality achieved when 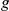 takes the locally optimal nonlinearity 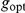 of Eq. (7). Here, for a standardized PDF 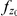 with zero mean and unity variance 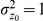, the scaled noise 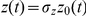 has its PDF 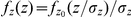 and the Fisher information satisfies 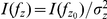 [1], [34]. It is known that a standardized Gaussian PDF 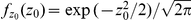 has the minimal Fisher information 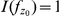 and any standardized non-Gaussian PDF 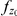 has the Fisher information 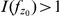 [2]. It can be seen that, the linear system 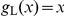 has its output signal-to-noise ratio 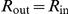 in Eq. (3). Thus, the output-input signal-to-noise ratio gain 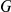 in Eq. (8) also clearly represents the expected performance improvement of the nonlinearity 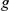 over the linear system 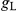.

### Optimum asymptotic efficacy for signal detection

Secondly, we consider the observation vector 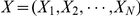 of real-valued components 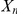 by
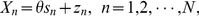
(9)where the components 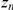 form a sequence of independent and identically distributed (i.i.d.) random variables with PDF 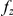, and the known signal components 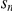 are with the signal strength 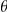 [6]. For the known signal sequence 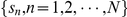, it is assumed that there exists a finite (non-zero) bound 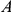 such that 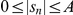, and the asymptotic average signal power is finite and non-zero, i.e. 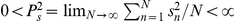 [6]. Then, the detection problem can be formulated as a hypothesis-testing problem of deciding a null hypothesis 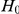 (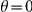) and an alternative hypothesis 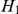 (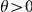) describing the joint density function of 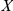 with
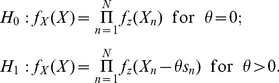
(10)Consider a generalized correlation detector
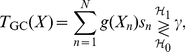
(11)where the memoryless nonlinearity 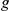 has zero mean under 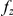, i.e. 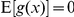 [6]. In the asymptotic case of 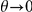 and 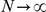, the test statistic 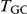, according to the central limit theorem, converges to a Gaussian distribution with mean 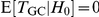 and variance 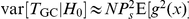 under the null hypotheses 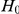 [6]. Using Eqs. (4) and (5), 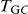 is asymptotically Gaussian with mean 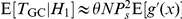 and variance 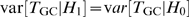 under the hypothesis 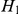 [6].

Given a false alarm probability 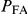, the asymptotic detection probability 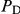 for the generalized correlation detector of Eq. (11) can be expressed as [2], [6]

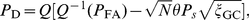
(12)with 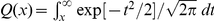 and its inverse function 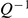 [2], [6]. Thus, for fixed 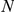 and 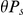 (since the signal is known), 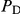 is a monotonically increasing function of the normalized asymptotic efficacy 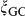 given by [6]

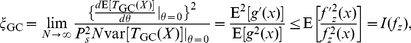
(13)with equality being achieved when 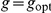 in Eq. (7). This result also indicates that the asymptotic optimal detector is just the locally optimal detector established by the Taylor expansion of the likelihood ratio test statistic 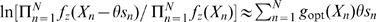 (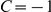) in terms of the generalized Neyman-Pearson lemma [2], [6].

Interestingly, with 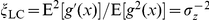 achieved by a linear correlation detector (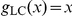 in Eq. (11)) as a benchmark [5], [6], the asymptotic relative efficiency
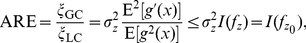
(14)provides an asymptotic performance improvement of a generalized correlation detector over the linear correlation detector when both detectors operate in the same noise environment [5], [6].

Next, consider the weak random signal components 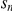 has PDF 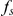 with zero mean 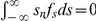 and variance 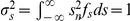 in the observation model of Eq. (9) [5], [6]. Here, the signal components 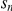 are i.i.d. Then, this random signal hypothesis test becomes [6]

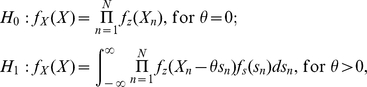
for determining whether the random signal is present or not. Consider a generalized energy detector
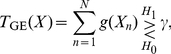
(15)where we also assume 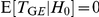, and then 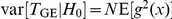. Furthermore, in the asymptotic case of 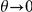, the expectation [6]

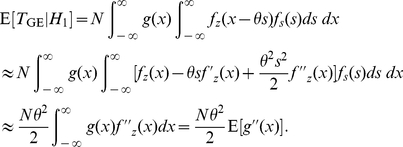
(16)Thus, the efficacy of a generalized energy detector is defined as [6]

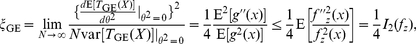
(17)where 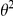 is treated as the signal strength parameter and 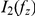 is the second order Fisher information [6], [7]. It is noted that the equality of Eq. (17) is achieved as 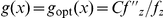 for a constant 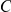 [6]. Given a false alarm probability 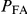, the asymptotic detection probability 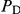 for the generalized energy detector of Eq. (15) is a monotonically increasing function of the efficacy 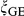 [5]–[7].

### Cross-correlation coefficient for signal transmission

Thirdly, we transmit a weak aperiodic signal 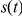 through the nonlinearity 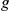 of Eq. (1) [13]. Here, the signal 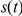 is with the average signal variance 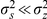, the zero mean and the upper bound A (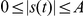). For example, 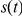 can be a sample according to a uniformly distributed random signal equally taking values from a bounded interval. The input cross-correlation coefficient of 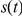 and 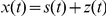 is defined as [2], [13]

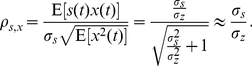
(18)Using Eqs. (4) and (5), the output cross-correlation coefficient of 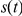 and 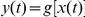 is given by
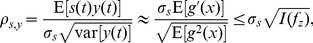
(19)which has its maximal value as 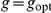 of Eq. (7). Then, the cross-correlation gain 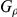 is bounded by
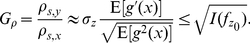
(20)


### Mean square error of an unbiased estimator

Finally, for the 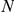 observation components 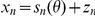, we assume the signal 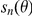 are with an unknown parameter 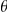. As the upper bound 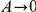 (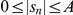), the Cramér-Rao inequality indicates that the mean squared error of any unbiased estimator of the parameter 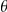 is lower bounded by the reciprocal of the Fisher information [1], [2] given by
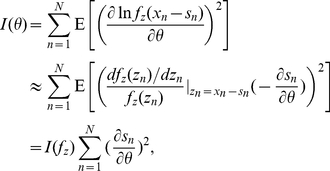
(21)which indicates that the minimum mean square error of any unbiased estimator is also determined by the Fisher information 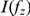 of a distribution, as 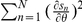 is given.

Therefore, just as the Fisher information represents the lower bound of the mean squared error of any unbiased estimator in signal estimation [1], [2], the physical significance of the Fisher information 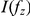
(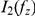) is that it provides a unified upper bound of the performance for locally optimal processing in the considered signal processing cases.

**Figure 1:**
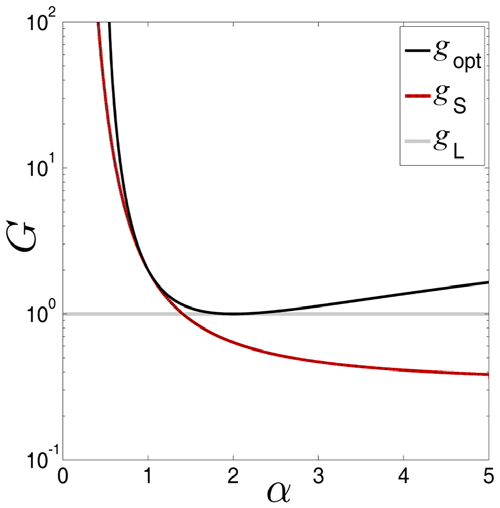
The output-input signal-to-noise ratio gain 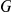
 **.** The output-input signal-to-noise ratio gain 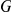 versus the exponential decay parameter 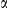 of the generalized Gaussian noise for the locally optimal nonlinearity 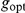 (solid line), the sign nonlinearity 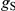 (red line) and the linear system 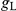 (dotted line), respectively.

Aiming to explain the upper bound of the performance for locally optimal processing as Fisher information, we here show an illustrative example in Fig. 1. Consider the generalized Gaussian noise with PDF
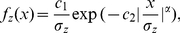
(22)where 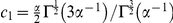, 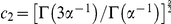 for a rate of exponential decay parameter 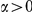 [2], [6]. The corresponding locally optimal nonlinearity is 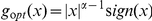 and the output-input signal-to-noise ratio gain in Eq. (8) is 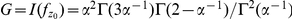 (solid line), as shown in Fig. 1. For comparison, we also operate the sign nonlinearity 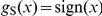 and the linear system 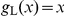 in the generalized Gaussian noise. The output-input signal-to-noise ratio gain in Eq. (8) of 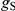 is 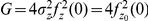 (dashed line), as shown in Fig. 1. For the linear system 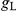, Eq. (8) indicates that 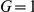 (dotted line) for 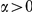, as plotted in Fig. 1. It is seen in Fig. 1 that, only for 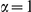, the performance of 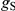 attains that of the locally optimal nonlinearity of 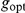. This is because, the nonlinearity 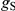 is just the locally optimal nonlinearity for Laplacian noise (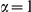), and the Fisher information limit 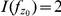 is achieved. Likewise, for Gaussian noise (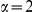), the linear system 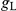 is optimal and the output-input SNR gain 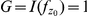. It is noted that the above analyses are also valid for the asymptotic relative efficiency of Eq. (14) and the cross-correlation gain of Eq. (20).

### Fisher information condition for stochastic resonance

Stochastic resonance (SR), being contrary to conventional approaches of suppressing noise, adds an appropriate amount of noise to a nonlinear system to improve its performance [8]–[32]. SR emerged from the field of climate dynamics [8], and the topic has flourished in physics [15]–[19] and neuroscience [13], [14], [20]. The notion of SR has been widened to include a number of different mechanisms [15], [17], [25], and SR effects have also been demonstrated in various extended systems [9]–[20], [25] and complex networks [21]–[24], [27].

An open question concerning SR is that, under the asymptotic cases of weak signal and large sample size, can SR play a role in locally optimal processing? Here, based on the Fisher information inequalities, we will demonstrate that SR is inapplicable to performance improvement for locally optimal processing.

For a given observation 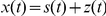, we add the extra noise 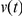, independent of the initial noise 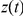 and the signal 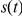, to 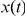. Then, the updated data 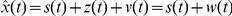. Here, the composite noise 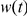 has a convolved PDF
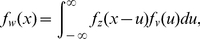
(23)where 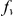 is the PDF of noise 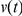. Currently, the weak signal 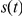 is corrupted by the composite noise 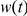, and then the performance measures of locally optimal processing in Eqs. (6), (13), (17), (19) and (21) should be replaced with 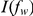 (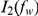). It can be shown by the Cauchy-Schwarz inequality that [34]

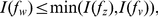
(24)

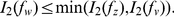
(25)This is because that, if 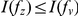, then using 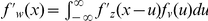 and the Cauchy-Schwarz inequality [34]

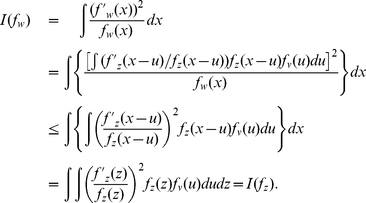
(26)Similarly, substituting 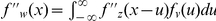 into Eq. (26), we also obtain 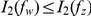 of Eq. (25).

Therefore, in asymptotic cases of weak signal and large sample size, Eqs. (24) and (25) show that SR cannot improve the performance of the above four locally optimal processing cases by adding more noise. However, the asymptotic limits of weak signal and large sample size are well delimited, and may not be met in practice. It is interesting to note that, under less restrictive conditions, noise-enhanced effects have been observed in fixed locally optimal detectors [9], suboptimal detectors [26], [29], the optimal detector with finite sample sizes [11] or non-weak signals [11], [25], soft-threshold systems [30] and the dead-zone limiter detector [28] by utilizing the constructive role of noise.

**Figure 2:**
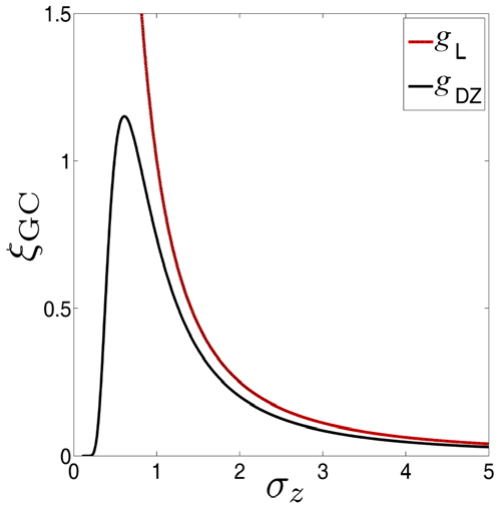
The normalized asymptotic efficacy 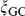
 **.** The normalized asymptotic efficacy 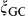 of the dead-zone limiter nonlinearity 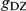 (solid line) and the linear system 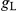 (red line) as a function of the RMS amplitude 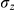 of Gaussian noise (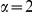).

We here present an illustrative example of SR that occurs outsides restrictive conditions, where a suboptimal detector is adopted for Gaussian noise. Consider a generalized correlation detector of Eq. (11) based on the dead-zone limiter nonlinearity
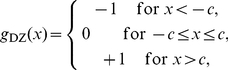
(27)with response thresholds at 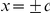 [6]. For the generalized Gaussian noise of Eq. (22), the normalized asymptotic efficacy 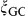 in Eq. (13) of 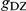 can be rewritten as
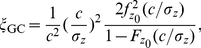
(28)where 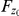 is the cumulative distribution function of the standardized generalized Gaussian noise PDF 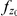 [28]. For a fixed response threshold 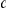 (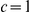 without loss of generality), we plot the the normalized asymptotic efficacy 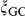 (solid line) of the dead-zone limiter nonlinearity 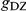 as a function of the RMS amplitude 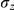 of Gaussian noise (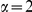), as shown in Fig. 2. It is clearly seen in Fig. 2 that the SR effect appears, and 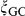 achieves its maximum 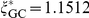 at a non-zero level of 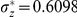. If the original Gaussian noise RMS 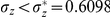, we can add independent Gaussian noise 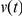 with its RMS amplitude 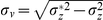 to increase 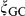 to the maximum 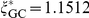 [28]. However, 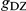 is a suboptimal nonlinearity for Gaussian noise, and the locally optimal detector is the linear correlation detector based on the linear system 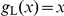 in Eq. (11). It is seen in Fig. 2 that 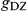 can not overperform 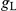 (dashed line), even we can add the appropriate amount of noise to exploit constructive role of noise in 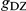.

## Discussion

In this paper, for a weak signal in additive white noise, it is theoretically demonstrated that the optimum performance for locally optimal processing is upper bounded by the Fisher information of the noise distribution, and this is uniformly obtained in (i) the maximum output signal-to-noise ratio ratio for a periodic signal; (ii) the optimum asymptotic efficacy for signal detection; (iii) the best cross-correlation coefficient for signal transmission; and (iv) the minimum mean square error of an unbiased estimator. Based on the Fisher information inequalities, it is demonstrated that SR cannot improve locally optimal processing under the usual conditions. However, outside these restrictive conditions of weak signal and large sample size, improvement by addition of noise through SR can be achieved, and becomes an attractive option for nonlinear signal processing. The analysis in the paper has focused on the simplest case of additive white noise as an essential reference, and an interesting extension for future work is to examine the affect of considering different forms of colored noise [15], [31], [32].

## Methods

Under the assumption of weak signals, the Taylor expansion of the noise PDF is utilized in Eqs. (4), (5), (16) and (21). The Cauchy-Schwarz inequality is extensively used in Eqs. (6), (13), (17), (19) and (26).
